# The role of a key transcription factor PU.1 in autoimmune diseases

**DOI:** 10.3389/fimmu.2022.1001201

**Published:** 2022-09-29

**Authors:** Yilong Fang, Weile Chen, Zhe Li, Yu Chen, Xuming Wu, Xiangling Zhu, Huihui Wang, Xiaochun Chen, Qiuni Liang, Jinghua Huang, Xintong Han, Wenming Hong, Xinming Wang, Wei Wei, Zhiying Yu, Jiajie Tu

**Affiliations:** ^1^ Institute of Clinical Pharmacology, Anhui Medical University, Key Laboratory of Anti-Inflammatory and Immune Medicine, Ministry of Education, Anhui Collaborative Innovation Center of Anti-Inflammatory and Immune Medicine, Hefei, China; ^2^ The First Clinical Medical College, Southern Medical University, Guangzhou, China; ^3^ Department of Gynecology, The First Affiliated Hospital of Shenzhen University, Health Science Center, Shenzhen Second People’s Hospital, Shenzhen, China; ^4^ The First Affiliated Hospital of Anhui Medical University, Hefei, China

**Keywords:** PU.1, autoimmune disease, RA, SLE, EAE

## Abstract

PU.1, a transcription factor member of the E26 transformation-specific family, affects the function of a variety of immune cells in several physiological and pathological conditions. Previous studies studying the role of PU.1 in pathological conditions have mainly focused on immune system-related cancers, and a series of articles have confirmed that PU.1 mutation can induce a variety of immune cell-related malignancies. The underlying mechanism has also been extensively validated. However, the role of PU.1 in other major immune system-related diseases, namely, systemic autoimmune diseases, is still unclear. It was only in recent years that researchers began to gradually realize that PU.1 also played an important role in a variety of autoimmune diseases, such as rheumatoid arthritis (RA), experimental autoimmune encephalomyelitis (EAE) and systemic lupus erythematosus (SLE). This review article summarizes the findings of recent studies that investigated the role of PU.1 in various autoimmune diseases and the related underlying mechanisms. Furthermore, it presents new ideas and provides insight into the role of PU.1 as a potential treatment target for autoimmune diseases and highlights existing research problems and future research directions in related fields.

## Introduction

PU.1 is essential for the differentiation and function of a variety of myeloid cells as a transcription factor (1, 2). Previous studies on PU.1 have focused on T cell-related cancers (3). Because PU.1 plays an essential role in the ontogenesis of a variety of immune cells (4–9) and various immune cell-associated tumors (3, 10, 11), researchers have begun to investigate the role of PU.1 in another large category of immune cell-related illnesses, systemic autoimmune diseases, which is a chronic and potentially life-threatening disease of the immune system that characterized by numerous autoantibodies and abnormal homeostasis of immune cells (12). Interestingly, multiple recent studies have shown that PU.1 is involved in the development of many autoimmune diseases, including rheumatoid arthritis (RA), experimental autoimmune encephalomyelitis (EAE) and systemic lupus erythematosus (SLE) and affects the pathogenic course of these diseases by influencing the function of a variety of immune cells.

In the current article, we review recent research progress in understanding the role of PU.1 in a variety of autoimmune diseases. Although PU.1 has been most extensively studied in RA, research is at an early stage and its role in RA remains controversial. Our research group was the first to confirm the pro-inflammatory effect of PU.1 on arthritis in PU.1 knockout mice. However, the specific role of PU.1 in different immune cells in RA appears to be different, which may explain the inconsistent results obtained by different research groups. In addition to RA, researchers have focused on the specific role of PU.1 in other autoimmune diseases, such as SLE and EAE. However, these studies are preliminary, and there is a lack of elegant *in vivo* models to conduct in-depth mechanistic studies. However, it is certain that PU.1 plays a key role in these autoimmune diseases, and this article summarizes the relevant literature and highlights future research directions.

## The role of PU.1 in autoimmune diseases

### PU.1 in rheumatoid arthritis

**Figure 1:**
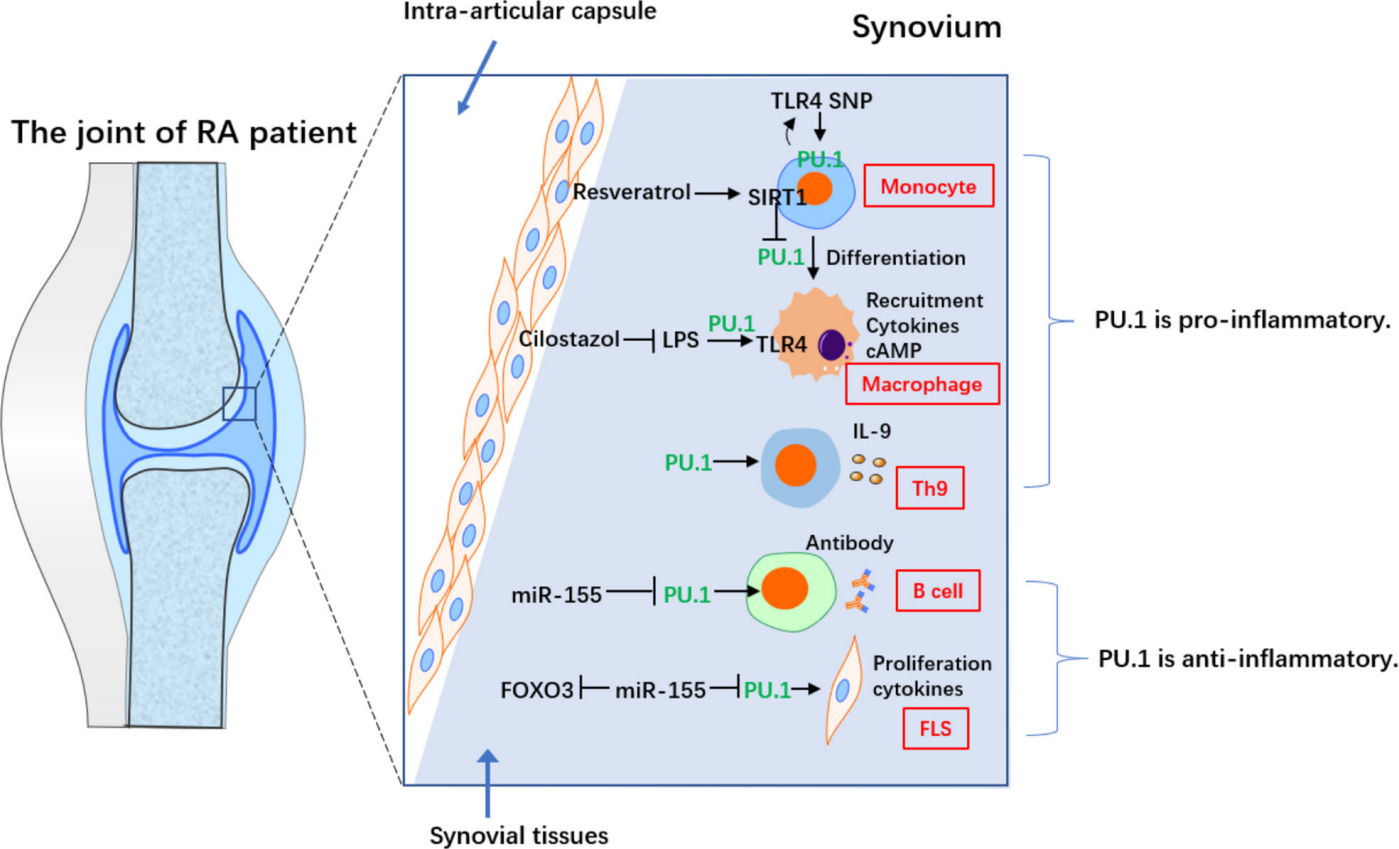
The contradictory role of PU.1 in different cells from RA. PU.1 acts as a pro-inflammatory factor in monocyte, macrophage and Th9 in RA. On the on contrary, PU.1 is an anti-inflammatory transcriptional factor in B cell and FLS in RA.

Researchers’ opinions regarding the role of PU.1 in RA is not conclusive (**Figure 1**). Interestingly, the anti-inflammatory effects of PU.1 in RA are associated with miR-155. MiR-155 highly expressed in peripheral blood B cells from RA patients, particularly in IgD^-^CD27^-^memory B cells from ACPA^+^ RA patients. The expression of miR-155 was also higher in the B cells of RA patients with synovial tissue containing ectopic germination centers compared with those of RA patients with diffuse synovial tissue. Stimulation of B cells from healthy donor with a series of pro-inflammatory cytokines promotes miR-155 and reduces PU.1. Blocking miR-155 in the B cells of patients with RA restored the PU.1 levels and reduced antibody production, suggesting that miR-155 is an important promoter of activation of B cell in RA (13). However, the expression of PU.1 in the peripheral blood mononuclear cells, synovial fluid and synovial tissue of RA patients, or a relationship between PU.1 and the production of B cell-derived antibodies were not detected in this study.

Another study reported that transfection of the PU.1 3’UTR repressed the TNF-α-induced production of IL-6 and IL-1β and downregulated miR-155 in RA-fibroblast-like synoviocytes (RA-FLS). In addition, transfection of the PU.1 3’UTR increased TNF-α-induced forkheadbox protein O3 (FOXO3) expression in RA-FLS, consistent with the effects of a miR-155 inhibitor. Furthermore, PU.1 and FOXO3 form a network of competitive endogenous RNAs (ceRNAs) by regulating miR-155. This study suggested that the PU.1 3’UTR regulates miR-155 activity by acting as a ceRNA for FOXO3, thereby attenuating TNF-α-induced proliferation and cytokine release by RA-FLS (14). However, the endogenous expression of PU.1 in RA-FLS was not detected. In addition to the production of inflammatory cytokines, RA-FLS also exhibit many other malignant features, such as invasion of bone and chondrocytes in the joint, promotion of differentiation from monocytes to osteoclasts, and production of MMPs, which require further research.

However, several studies reported that PU.1 also exerts a pro-inflammatory role in RA, which is often associated with the Toll-like receptor 4 (TLR4) pathway. TLR4 is an innate immune receptor that is predominantly expressed in myeloid cells. Cilostazol, which increases the intracellular cAMP levels, inhibited the lipopolysaccharide (LPS)-induced in TLR4 by repressing the transcriptional activity of PU.1 in macrophages from patients with RA. Furthermore, it inhibited the LPS-induced myeloid differentiation factor 88 (MyD88) and suppressed IκBα degradation and p65 nuclear translocation. In addition, it also inhibited the LPS-induced TNF-α and IL-1β, while inhibiting NF-κB pathway. Furthermore, cilostazol induced anti-inflammatory IL-10. In collagen antibody-induced arthritis (CAIA) model, treatment with cilostazol resulted in a down-regulation in the expression of TLR4 in the knee joint, which was associated with reduced macrophage recruitment. Thus, cilostazol treatment significantly suppressed synovial inflammation by repressing PU.1 expression in macrophages (15).

Monocyte-macrophage differentiation is essential in the pathophysiology of RA, as it leads to the secretion of multiple cytokines. It is, therefore, considered a potential clinical therapeutic target. Resveratrol, a SIRT1 activator, inhibited phorbol 12-myristate 13-acetic acid (PMA)-induced adhesion of monocyte from patients with RA, which could be prevented by sirtinol (SIRT1 inhibitor). In addition, resveratrol repressed the PMA-stimulated expression of macrophage markers and activation of NF-κB pathway, thereby inhibiting pro-inflammatory cytokines. In SIRT1 transgenic (Tg) mice, monocyte-macrophage differentiation, and activation of NF-κB pathway, as well as the expression of proinflammatory cytokines were inhibited compared to those in control mice. Interestingly, resveratrol-induced SIRT1 repressed phosphorylation and nuclear translocation of PU.1, thereby inhibiting monocyte differentiation. To summarize, SIRT1 blocks monocyte differentiation by inhibiting PU.1 and related inflammatory signaling, suggesting a critical role for the macrophage-specific SIRT1-PU.1 regulatory axis in regulating RA synovial inflammation (16).

Researchers have presented functional data for the single nucleotide polymorphism (SNP) rs7873784 in the 3’-UTR of the *TLR4*. The 3’-UTR of TLR4 activated the promoter of TLR4 in the U937 cells (a human monocytic cell line). In addition, the minor rs7873784(C) allele created a binding site for PU.1. Increased PU.1 binding further enhanced transcription of TLR4. This functional PU.1 loci might induce TLR4 in individuals with this rs7873784 minor C variant and regulate related autoimmune disease, including RA (17).

Th9 has been shown to be abundantly expressed in peripheral blood, synovial fluid, and synovial membrane of RA patients. Th9 frequency positively correlated with DAS28 and with the degree of histological organization of B and T cells in ectopic lymphoid structures (18). As a newly discovered CD4^+^ T cell subset in recent years, Th9 can specifically expressed PU.1 and IL-9. Research has confirmed that PU.1 directly binds to the promoter region of IL-9. PU.1 is essential for the development of Th9-relatead inflammation (19). In another study, PBMCs were collected from 55 RA outpatients (15 drug-free, 20 successfully treated with infliximab, and 20 with poor response to infliximab) and 10 controls and cultured with or without infliximab. Unstimulated RA patients had the highest percentage of Th9 cells in the unstimulated condition. The percentages of Th9, CCR7^+^CD45RA^+^ (central memory), and CCR7^-^CD45RA^+^ (effector memory) cells expressing PU.1 and IRF4 were increased in the poor response group after stimulation with infliximab, but there are no changes after treatment of infliximab biosimilars. Th9 cells appear to be involved in immune responses to branded infliximab, but not to biosimilar infliximab epitopes, which may depend on the central and effector memory cells (20). Therefore, PU.1 and Th9 play an important role in RA inflammation, and blocking IL-9 may be a new idea for the treatment of RA.

### PU.1 in autoimmune encephalitis

**Figure 2:**
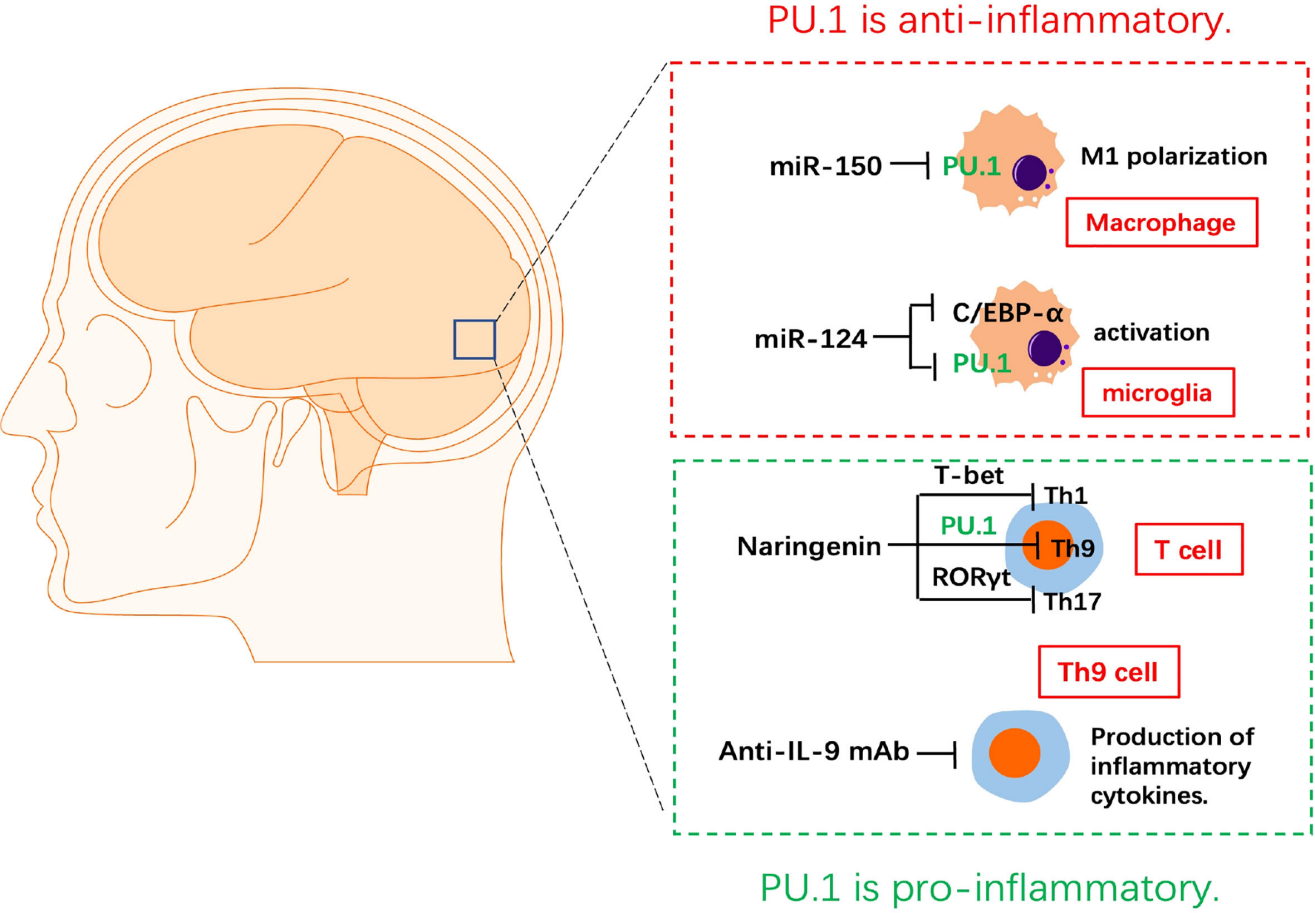
The role of PU.1 in experimental autoimmune encephalitis. MiRNA-150 reduces the production of pro-inflammatory cytokines and switches macrophage polarization from the M1 to the M2 *via* targeting PU.1. miR-124 represses the monocyte-macrophage activation *via* repressing C/EBP-α and PU.1 in microglia. Naringenin is a potential medicine in the treatment of EAE by inhibiting the Th1(T-bet), Th9(PU.1) and Th17(RORγt) differentiation of CD4^+^ T cells. Blocking IL-9 with anti-IL-9 mAb inhibits the development of EAE.

Researchers have studied the role of PU.1 and its targeted miRNAs in mice with experimental autoimmune encephalitis (EAE) and primary macrophages from the central nervous system (CNS) (**Figure 2**). PU.1 was highly expressed in the spinal cord of EAE mice and promoted M1 polarization of macrophages, which was negatively associated with expression of miR-150 in the chronic phase of EAE. A luciferase reporter assay confirmed the direct binding between PU.1 3’UTR and miR-150. Up-regulation of miRNA-150 in macrophages reduces the production of pro-inflammatory cytokines and switches macrophage polarization from the M1 to the M2 (21).

MiR-124 is specifically expressed in microglia, but not in peripheral-derived macrophages. Up-regulation of miR-124 directly inhibits the CCAAT/enhancer binding protein-α (C/EBP-α) and PU.1 in macrophages, causing these cells to transition from an activated phenotype to a quiescent CD45^low^MHCII^low^ phenotype similar to that of resting microglia. Peripheral administration of miR-124 leads to inactivation of macrophages in EAE, decreased activation of myelin-specific T-cells, and significant inhibition of EAE. Therefore, miR-124 is an essential miRNA of resting microglia in the CNS and a previously unknown regulator of monocyte-macrophage activation *via* repressing C/EBP-α and PU.1 (22).

Th9 can induce EAE independently, CNS lesions in Th9 cell recipients were characterized by massive and more evenly distributed parenchymal infiltrates associated with marked demyelination (23). IL-9 blockade with anti–IL-9 mAb inhibited the development of EAE. The protective effect of IL-9 blockade in EAE was likely mediated *via* inhibition of the development of MOG peptide-specific T cells (24). Anti–IL-9 mAb treatment may provide an effective therapeutic strategy against autoimmune diseases. Naringenin is a flavonoid found in citrus fruits. It has anti-inflammatory and antioxidant functions. Naringenin was found to reduce and delay the incidence of EAE and alleviate its symptoms, accompanied by a decrease in demyelination in the spinal cord and infiltration of immune cells. In addition, naringenin repressed the levels of the pro-inflammatory CD4^+^ T cell subgroups Th1, Th9, and Th17 cells and their transcription factors T-bet, PU.1, and RORγt in the CNS and lymph nodes of EAE mice. However, narigenin didn’t affect the anti-inflammatory CD4^+^ T cells subgroups Th2 and regulatory T cell (Treg) in the CNS or lymph nodes. Naringenin is a potential medicine in the treatment of EAE by regulating the differentiation of CD4^+^ T cell subtypes (25).

Overall, PU.1 plays a role in promoting EAE development by activating M1 macrophages and Th9 cells. The construction of EAE models using macrophages (LysM-cre)- and Th9 cell (IL9-cre)-specific PU.1 KO or KI mice, could further increase the understanding of the specific mechanism of PU.1 in the pathogenesis of EAE.

### PU.1 in systemic lupus erythematosus

**Figure 3:**
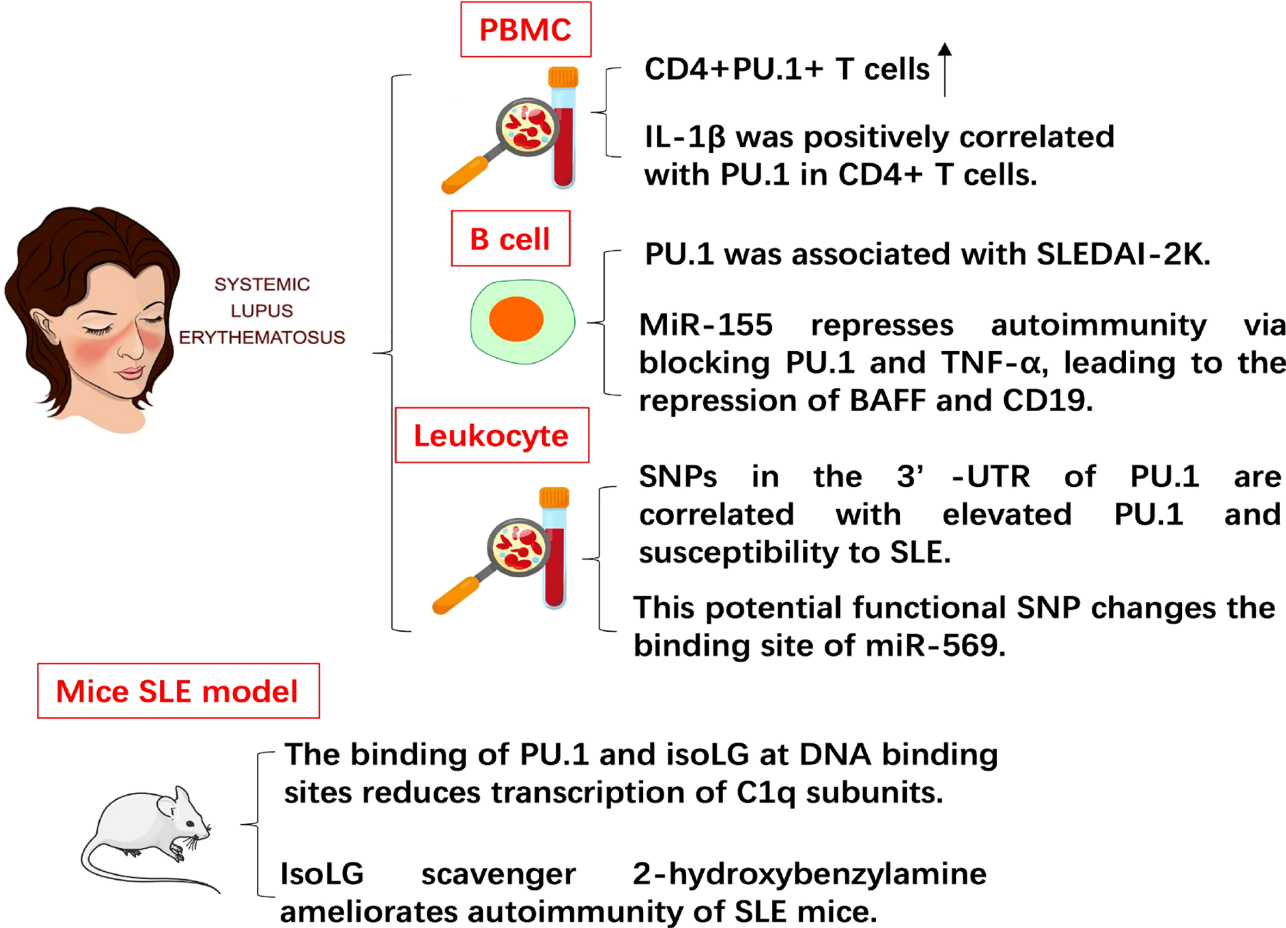
The role of PU.1 in SLE. The expression of PU.1 is positively correlated with IL-1 in CD4+ T cells from patients with SLE. PU.1 is also positively associated with SLE disease activity index 2 K (SLEDAI-2K). SNP (rs1057233) in the 3’-UTR of PU.1 is correlated with elevated PU.1 and susceptibility to SLE. In mouse SLE model, the binding of PU.1 and isoLG at DNA binding sites reduces transcription of C1q subunits. IsoLG scavenger ameliorates autoimmunity of SLE mice.

SLE is a multisystem autoimmune disease (**Figure 3**). The ratio of CD4^+^PU.1^+^ T cells in the PBMCs of SLE patients increased compared with that in the PBMCs of healthy controls. Patients with SLE had higher plasma concentrations of pro-inflammatory cytokines than healthy controls. The level of plasma TGF-β1 in patients with SLE decreased compared with that in healthy controls. In addition, the level of IL-1β was positively correlated with the expression of PU.1 in CD4^+^ T cells in SLE patients. This study was the first to assess the expression pattern of PU.1 in CD4^+^ T cells from patients with SLE, implying that PU.1 might be involved in SLE pathogenesis (26).

MiR-155, which is essential in the pathogenesis of SLE, has been proved in modulating activation and survival of B cell. PU.1 increased in the PBMCs and B cells of pediatric patients with SLE (pSLE). The level of PU.1 was associated with the SLE disease activity index 2 K (SLEDAI-2K). Overexpression of miR-155 or down-regulation of PU.1 inhibited the expression of BAFF and TNF-α. Up-regulation of miR-155 also reduced the percentage of BAFF^+^ B cells and CD19. To sum up, miR-155 represses autoimmunity *via* blocking PU.1 and TNF-α, leading to the repression of BAFF and CD19 (27).

Significant associations were detected in two SNPs (rs10769258 and rs4752829) in intron 2 of PU.1. Another potential functional SNP (rs1057233) in 3’-UTR of PU.1 was identified in strong linkage disequilibrium with the SNPs in intron 2 of PU.1. The number of risk alleles at rs1057233 was closely associated with the expression levels of PU.1. This potential functional SNP also changed the binding site of miR-569. Furthermore, miR-569 repressed the expression of reporter constructs that contained the non-risk allele but did not contain the 3’-UTR sequence of the risk allele. These results of this study suggest that SNPs in the 3’-UTR of PU.1 are correlated with elevated expression of PU.1 mRNA and susceptibility to SLE (28). Isolevuglandin-adducted proteins (isoLG adducts) in monocytes and dendritic cells were significantly enriched in SLE patients and mouse SLE model. In addition, isoLG ligation of the transcription factor PU.1 at a critical DNA binding site markedly reduced transcription of all C1q subunits. Treatment of SLE mice with the specific isoLG scavenger 2-hydroxybenzylamine (2-HOBA) ameliorated parameters of autoimmunity, including plasma cell expansion, circulating IgG levels, and anti-dsDNA antibody titers (29). This result also indicates the potential pathogenic role of PU.1 in SLE.

Compared with healthy controls, SLE patients had significantly higher plasma IL-9 concentrations and mRNA levels, as well as an increased percentage of Th9 cells. In addition, serum IL-9 levels and Th9 percentage were correlated with SLE Disease Activity Index (SLEDAI). After methylprednisolone treatment, the percentages of Th9 cells and serum IL-9 levels decreased (30). The transcription regulator Bach2 is another potential target for SLE therapy, and Bach2 overexpression significantly inhibited the levels of PU.1, IRF4, IL-9, and Th9 in CD4^+^ T cells from SLE patients (31), implying that elevated IL-9 and Th9 levels may trigger the inflammatory process in SLE.

Similar to EAE, PU.1 also promotes excessive immune activation in SLE. Interestingly, miR-155 directly inhibits PU.1 in both RA and SLE. However, while miR-155 promotes activation and antibody production of B cell in RA, its expression decreases and B cell activation is repressed in SLE. This opposite effect of the miR-155-PU.1 axis on B cells in RA and SLE warrants further investigation.

### PU.1 in other autoimmune diseases

A significant up-regulation in the mRNA expression of PU.1 was detected in the inflammatory retina. Most PU.1^+^ cells co-localized with CD11c and F4/80 (macrophage markers) in retina. Knockdown of PU.1 significantly inhibited interphotoreceptor retinoid-binding protein-stimulated secretion of IFN and IL-2 in the lymph nodes. Thus, PU.1 might involve in the progression of experimental autoimmune uveoretinitis (EAU) inflammation (32). Researchers have analyzed binding profile of PU.1 in neutrophils from dozens of volunteers. The variants associated with different binding sites PU.1 were the molecular basis for genetically-induced cellular differences and susceptibility to autoimmune diseases. This also provides functional explanations for 27 genes with potential immune signatures. This study demonstrates that PU.1 and its target enhancers are involved in neutrophil-related transcriptional control and susceptibility to immune disease (33).

Another study demonstrated that the Ikaros family transcription factor IKZF3 (Aiolos) increased during the maturation of mouse eosinophil lineage (34). Aiolos-deficient murine eosinophils exhibited changes in signaling pathways related to extracellular matrix organization, granulocyte-mediated immunity, degranulation and ERK/MAPK signaling. The promoter regions of these genes were rich in binding sites of Aiolos. Furthermore, global Aiolos deficiency decreased the proportion of eosinophils in peripheral tissues during the steady state, whereas a chimeric mouse model showed that the eosinophilic commitment is dependent on Aiolos. Aiolos deficiency repressed eosinophilic CCR3, ERK1/2 signaling, and CCL11-induced actin polymerization. Moreover, Aiolos expression was correlated to active chromatin marks enriched for binding sites of IKZF3, PU.1, and GATA-1 by analyzing ChIP-seq data of eosinophil-specific histone peaks. Therefore, the eosinophil homing to allergic states during homeostasis and inflammation is Aiolos-dependent (34).

In patients with ulcerative colitis, PU.1 expression and IL-9-secreting T cells increased. In animal models of colitis, IL-9 deficiency suppressed both acute and chronic colitis, mice with PU.1 deficient in T cells were protected from colitis, and treatment with an anti-IL-9 antibody suppressed colitis (35). Therefore, Th9 subsets play an important role in driving ulcerative colitis by regulating intestinal epithelial cells and may be a therapeutic target for chronic intestinal inflammation.

In immune-related pancytopenia (IRP) patients, the ratio of Th1/Th2 cells increased. Th9 belongs to one of the newly discovered Th2 cell subsets, which can specifically express PU.1 and IL-9. The ratio of Th9 cells among CD3^+^CD4^+^ cells and the levels of IL-9 in the untreated group were higher compared with those in the remission and control groups, and those of the remission group were higher than those in the control group. The percentage of Th9 cells among CD3^+^CD4^+^ cells and the expression of IL-9 were negatively associated with hemoglobin levels, red blood cell, white blood cell, platelet counts, and positively associated with the percentages of CD19^+^ B cells. The mRNA levels of PU.1 and BATF in IRP patients were higher compared with those in controls. The peripheral blood Th9 cell percentage and serum IL-9 are elevated in patients with IRP, which correlates with disease severity (36).

Natural resistance-associated macrophage protein 1 (Nramp1) is a proton/divalent cation reverse transporter that is expressed only in monocytes/macrophages and is important in innate resistance to phagocytic pathogens. In humans, it has been linked to autoimmune diseases, such as RA and Crohn’s disease. Researchers have demonstrated that restricted expression of Nramp1 is mediated by IRF-8 (macrophage-specific transcription factor). The myc interaction zinc finger protein 1 (Miz-1) was identified as a novel interacting protein of IRF-8 *via* yeast double hybrid screening. This interaction was limited to immune cells and occurred in the Nramp1 promoter in a PU.1-dependent manner. In addition, IRF-8 knockout mice were sensitive to a range of pathogens (37).

Researchers have identified a new circRNA, circSnx5, which plays a central role in regulating dendritic cell (DC)-driven immunity and tolerance. Overexpression of circSnx5 inhibited DC activation and promoted the development of DC tolerance, whereas circSnx5 knockdown promoted activation of DC and inflammation. This circRNA acted as a miR-544 sponge to inhibit miRNA-mediated target inhibition of cytokine signaling 1 (SOCS1) and repress nuclear translocation of PU.1. In addition, heterogeneous ribonucleoprotein (hnRNP) C was identified as a key splicing factor (SF) for the production of circSnx5 in DC. These data showed that vaccination with DCs under circSnx5 conditions prolongs the survival of allogeneic grafts of the heart in mice and reduces experimental autoimmune myocarditis (38).

MiR-148a is a key regulator of differentiation of monocyte-derived DC (moDC). The deficiency of miR-148a impaired the development of moDCs. Mechanistic studies proved that MAFB (a transcription factor that hinders moDC differentiation) is a target of miR-148a. It further confirmed that PU.1 could activate miR-148a, which is critical for the generation of moDCs. Inhibition of miR-148a repressed the inhibitory effect of PU.1 on MAFB. In addition, an increase in miR-148a levels was observed in the monocytes of patients with psoriasis. Intradermal injection of antagomir-148a, or miR-148a deficiency, greatly inhibited the psoriasis-like symptoms in a mouse model of psoriasis. To sum up, this study identifies the key function of the PU.1-miR-148a-MAFB axis in differentiation of moDC and propose a potential therapeutic pathway in psoriasis (39).

Another study showed that the IL-4 and TGF-β1 levels were elevated in allergic rhinitis (AR) patients compared with those in healthy controls. The IL-9, PU.1, IRF4, and Th9 cell counts in AR patients were also significantly elevated compared with those in healthy controls. In addition, IL-9 levels were positively correlated with EOS expression, RQLQ, and VAS scores (40).

In conclusion, PU.1 has been demonstrated to activate autoimmunity and promote inflammation in some common autoimmune diseases, including RA, SLE, EAE, etc. PU.1 can affect the function of a variety of immune cells in these diseases (**Table 1**).

**Table 1:** The role of PU.1 in other autoimmune diseases.

Disease	PU.1’s role	Related cells	Related pathways	Ref
experimental autoimmune uveoretinitis (EAU)	Knockdown of PU.1 inhibited IRBP-stimulated secretion of IFN and IL-2.	Macrophage	None	(17)
Autoimmune diseases, such as RA and IBD	The variants associated with different binding sites PU.1 were the molecular basis for genetically-induced cellular differences and susceptibility to autoimmune diseases. PU.1 is a key regulator of the phenotype of IL-9-secreting T cells and is important for the development of inflammation.	Neutrophil Th9	None None	(18–20, 23, 24, 29–31, 35, 41, 42)
Eosinophil lineage-related inflammation	Aiolos expression was correlated to active chromatin marks enriched for binding sites of PU.1.	eosinophil	ERK/MAPK pathway	(20)
Immune-related pancytopenia	The expression of PU.1 in IRP patients were higher compared with those in controls.	T cell	none	(22)
Autoimmune diseases, such as RA and Crohn’s disease	Restricted expression of Nramp1 is mediated by IRF-8. Miz-1 was identified as a interacting protein of IRF-8. This interaction occurred in the Nramp1 promoter in a PU.1-dependent manner.	monocyte/macrophage	none	(25)
Experimental autoimmune myocarditis (EAM)	CircSnx5 inhibited DC activation and promoted the development of DC tolerance. This circRNA acted as a miR-544 sponge to inhibit miRNA-mediated target inhibition of SOCS1 and repress nuclear translocation of PU.1.	dendritic cell	none	(26)
Psoriasis	PU.1-miR-148a-MAFB axis is a potential therapeutic pathway in psoriasis.	monocyte-derived DC	none	(27)
Allergic rhinitis (AR)	The IL-9, PU.1, IRF4, and Th9 cell counts in AR patients are elevated in patients with allergic rhinitis compared with those in healthy controls.	PBMC	none	(28)

## Conclusions and future perspectives

Although research on the role of PU.1 in autoimmune diseases is at an early stage, the important role of PU.1 in these diseases is unquestionable. The relevant literature can be divided into three phases. In the first stage, researchers confirmed that PU.1 is a key transcription factor involved in the normal development of a variety of myeloid cells, suggesting its role as a potential therapeutic target for autoimmune diseases. In the second stage, more attention was paid to the role of PU.1 in myeloid cell-associated cancers and related mechanisms, and key mutations in PU.1, which could cause AML, were identified. In the third phase, in recent years, researchers have shifted their focus to the role of PU.1 in autoimmune diseases, including RA, SLE, and EAE. In addition, Th9 has also been widely studied, which further indicates that PU.1 and Th9 may serve as therapeutic targets for these diseases.

The role of PU.1 has been widely studied in RA. However, if it has pro-inflammatory or anti-inflammatory effects on RA remains to be elucidated. Furthermore, PU.1 appears to exhibit different functions in different cells. Although it exerts an anti-inflammatory effect on B cells, it promotes a pro-inflammatory phenotype in macrophages. Both Th9 and IL-9 currently exhibit pro-inflammatory effects in RA, SLE, and EAE, and blocking IL-9 can be effectively alleviated. These results suggest that Th9/IL-9 is a potential therapeutic target for autoimmune diseases (18, 41, 42). A study demonstrates the pro-inflammatory role of IL-9 in CIA, and *in vivo* injection of anti-IL-9 mAb can effectively alleviate joint inflammation, which is associated with a decrease in CD4^+^ TNF-α^+^ cells and an increase in CD4^+^ FoxP3^+^ IL-10^+^ cells. This study provides a basis for future research on PU.1 and Th9 in RA (43). However, none of these studies confirmed the role of PU.1 *in vivo*. Our research group used PU.1 knockout mice to construct a CAIA model and demonstrated for the first time that PU.1 exhibited pro-inflammatory effects *in vivo*. More importantly, a small molecule inhibitor of PU.1 could significantly inhibit arthritis symptoms in CAIA mice. The expression of PU.1 in the joint synovium of RA patients was also significantly upregulated. These results suggest that PU.1 is likely to function as a pro-inflammatory transcription factor in RA and that it is a potential RA treatment target.

Based on the existing literature, PU.1 has been demonstrated to activate autoimmunity and promote inflammation in some autoimmune diseases, including RA, SLE, EAE, and AR. Furthermore, PU.1 can affect the function of a variety of immune cells in these diseases. In RA, PU.1 can affect the function of B cells, FLS, and macrophages, whereas in EAE, PU.1 mainly promotes disease progression by acting on macrophages and T cells. In SLE, PU.1 is associated with excessive activation of T cells and B cells, while in other autoimmune diseases, in addition to the above-mentioned immune cells, PU.1 also plays a role in other immune cells such as neutrophils, eosinophils, and DCs. These studies illustrate that PU.1 can be expressed in a variety of immune cells and that it affects the function of these immune cells, influencing the course of related diseases. In the future, we need to use cell-specific PU.1 knockout mice to further investigate the specific role and underlying mechanism of PU.1 in these cells. Furthermore, we need to identify the cells in which PU.1 plays a major regulatory role, affecting the development of different diseases, which is important for the development of relevant drug targets in the future. Moreover, PU.1 promotes the differentiation of Th9 cells. In PU.1-related autoimmune diseases, Th9 cells and their secreted cytokine, IL-9, both exhibit abnormal activation. The relationship between PU.1 and Th9 was confirmed in RA, EAE, SLE, and AR. PU.1 is closely related to these autoimmune diseases by regulating the abnormal activation of Th9, suggesting that modulation of PU.1 in Th9 cells is a potential research direction that deserves further exploration.

## Author contributions

YF, WC, YC and JT drafted the manuscript. ZL, XuW, XZ, HW, XC, QL, JH, XH, WH, XiW, WW and ZY revised the manuscript. All authors contributed to the article and approved the submitted version.

## Funding

This work was supported by the project of improvement of scientific ability of Anhui Medical University(2020xkjT009), Shenzhen Science and Technology Innovation Committee (JCYJ20210324102806018 and JCYJ20210324103001003); Sanming Project of Medicine in Shenzhen (SZSM201812041); Clinical Research Funding from Shenzhen Second People’s Hospital (20203357030).

## Conflict of interest

The authors declare that the research was conducted in the absence of any commercial or financial relationships that could be construed as a potential conflict of interest.

## Publisher’s note

All claims expressed in this article are solely those of the authors and do not necessarily represent those of their affiliated organizations, or those of the publisher, the editors and the reviewers. Any product that may be evaluated in this article, or claim that may be made by its manufacturer, is not guaranteed or endorsed by the publisher.

## References

[1] Hosokawa H Rothenberg EV. How transcription factors drive choice of the T cell fate. Nat Rev Immunol (2021) 21:162–76. doi: 10.1038/s41577-020-00426-6 PMC7933071 32918063

[2] Solomon LA Podder S He J Jackson-Chornenki NL Gibson K Ziliotto RG. Coordination of myeloid differentiation with reduced cell cycle progression by PU . 1 induction of MicroRNAs targeting cell cycle regulators and lipid anabolism. Mol Cell Biol (2017) 37:1–17. doi: 10.1128/MCB.00013-17 PMC5477546 28223367

[3] Burda P Laslo P Stopka T. The role of PU.1 and GATA-1 transcription factors during normal and leukemogenic hematopoiesis. Leukemia (2010) 24:1249–57. doi: 10.1038/leu.2010.104 20520638

[4] Guanglan LI Wenke HAO Wenxue HU. Transcription factor PU.1 and immune cell differentiation (Review). Int J Mol Med (2020) 46:1943–50. doi: 10.3892/ijmm.2020.4763 33125129

[5] Carotta S Wu L Nutt SL. Surprising new roles for PU.1 in the adaptive immune response. Immunol Rev (2010) 238:63–75. doi: 10.1111/j.1600-065X.2010.00955.x 20969585

[6] Yoon H Jeremy M. Boss. PU.1 binds to a distal regulatory element that is necessary for b-cell specific expression of the class II transactivator. J Immunol (2010) 184:5018–28. doi: 10.4049/jimmunol.1000079.PU.1 PMC3472449 20363966

[7] Joo M Kwon M Azim AC Sadikot RT Timothy S Christman JW. Genetic determination of the role of PU.1 in macrophage gene expression. Biochem Biophys Res Commun (2008) 372:97–102. doi: 10.1016/j.bbrc.2008.04.189 18485892 PMC2494535

[8] Yashiro T Takeuchi H Nakamura S Tanabe A Hara M Uchida K. PU.1 plays a pivotal role in dendritic cell migration from the periphery to secondary lymphoid organs *via* regulating CCR7 expression. FASEB J (2019) 33:11481–91. doi: 10.1096/fj.201900379RR 31314592

[9] Rothenberg EV Hosokawa H Ungerbäck J. Mechanisms of action of hematopoietic transcription factor PU.1 in initiation of T-cell development. Front Immunol (2019) 10:228. doi: 10.3389/fimmu.2019.00228 30842770 PMC6391351

[10] Kastner P Chan S. PU.1: A crucial and versatile player in hematopoiesis and leukemia. Int J Biochem Cell Biol (2008) 40:22–7. doi: 10.1016/j.biocel.2007.01.026 17374502

[11] Moreau-Gachelin F. Spi-1/PU.1: an oncogene of the ets family. BBA - Rev Cancer (1994) 1198:149–63. doi: 10.1016/0304-419X(94)90011-6 7819272

[12] Shapira Y Agmon-Levin N Shoenfeld Y. Geoepidemiology of autoimmune rheumatic diseases. Nat Rev Rheumatol (2010) 6:468–76. doi: 10.1038/nrrheum.2010.86 20567251

[13] Alivernini S Kurowska-Stolarska M Tolusso B Benvenuto R Elmesmari A Canestri S. MicroRNA-155 influences b-cell function through PU.1 in rheumatoid arthritis. Nat Commun (2016) 7. doi: 10.1038/ncomms12970 PMC5052655 27671860

[14] Xie Z Qu Y Shen P Wang B Wei K Du B. PU.1 attenuates TNF-α-induced proliferation and cytokine release of rheumatoid arthritis fibroblast-like synoviocytes by regulating miR-155 activity. Mol Med Rep (2018) 17:8349–56. doi: 10.3892/mmr.2018.8920 29693176

[15] Park SY Lee SW Baek SH Lee CW Lee WS Rhim BY. Suppression of PU.1-linked TLR4 expression by cilostazol with decrease of cytokine production in macrophages from patients with rheumatoid arthritis. Br J Pharmacol (2013) 168:1401–11. doi: 10.1111/bph.12021 PMC3596645 23072581

[16] Park SY Lee SW Kim HY Lee SY Lee WS Hong KW. SIRT1 inhibits differentiation of monocytes to macrophages: amelioration of synovial inflammation in rheumatoid arthritis. J Mol Med (2016) 94:921–31. doi: 10.1007/s00109-016-1402-7 26956118

[17] Korneev KV Sviriaeva EN Mitkin NA Gorbacheva AM Uvarova AN Ustiugova AS. Minor c allele of the SNP rs7873784 associated with rheumatoid arthritis and type-2 diabetes mellitus binds PU.1 and enhances TLR4 expression. Biochim Biophys Acta - Mol Basis Dis (2020) 1866. doi: 10.1016/j.bbadis.2019.165626 31785408

[18] Ciccia F Guggino G Rizzo A Manzo A Vitolo B La MMP. Potential involvement of IL-9 and Th9 cells in the pathogenesis of rheumatoid arthritis. Rheumatol (United Kingdom) (2015) 54:2264–72. doi: 10.1093/rheumatology/kev252 26178600

[19] Chang HC Sehra S Goswami R Yao W Yu Q Stritesky GL. The transcription factor PU.1 is required for the development of IL-9-producing T cells and allergic inflammation. Nat Immunol (2010) 11:527–34. doi: 10.1038/ni.1867 PMC3136246 20431622

[20] Talotta R Berzi A Doria A Batticciotto A Ditto MC Atzeni F. The immunogenicity of branded and biosimilar infliximab in rheumatoid arthritis according to Th9-related responses. Int J Mol Sci (2017) 18. doi: 10.3390/ijms18102127 PMC5666809 29023386

[21] Shakerian L Ghorbani S Talebi F Noorbakhsh F. MicroRNA-150 targets PU.1 and regulates macrophage differentiation and function in experimental autoimmune encephalomyelitis. J Neuroimmunol (2018) 323:167–74. doi: 10.1016/j.jneuroim.2018.06.010 30196828

[22] Ponomarev ED Veremeyko T Barteneva N Krichevsky AM Weiner HL. MicroRNA-124 promotes microglia quiescence and suppresses EAE by deactivating macrophages. Via C/EBP-α-PU.1 Pathway Nat Med (2011) 17:64–70. doi: 10.1038/nm.2266 PMC3044940 21131957

[23] Jäger A Dardalhon V Sobel RA Bettelli E Kuchroo VK. Th1, Th17, and Th9 effector cells induce experimental autoimmune encephalomyelitis with different pathological phenotypes. J Immunol (2009) 183:7169–77. doi: 10.4049/jimmunol.0901906 PMC2921715 19890056

[24] Li H Nourbakhsh B Ciric B Zhang G-X Rostami A. Neutralization of IL-9 ameliorates experimental autoimmune encephalomyelitis by decreasing the effector T cell population. J Immunol (2010) 185:4095–100. doi: 10.4049/jimmunol.1000986 PMC2978501 20805418

[25] Wang J Qi Y Niu X Tang H Meydani SN Wu D. Dietary naringenin supplementation attenuates experimental autoimmune encephalomyelitis by modulating autoimmune inflammatory responses in mice. J Nutr Biochem (2018) 54:130–9. doi: 10.1016/j.jnutbio.2017.12.004 29331869

[26] Xiang N Fang X Sun XG Zhou YB Ma Y Zhu C. Expression profile of PU.1 in CD4+T cells from patients with systemic lupus erythematosus. Clin Exp Med (2021) 21:621–32. doi: 10.1007/s10238-021-00717-9 33966135

[27] Aboelenein HR Hamza MT Marzouk H Youness RA Rahmoon M Salah S. Reduction of CD19 autoimmunity marker on b cells of paediatric SLE patients through repressing PU.1/TNF-α/BAFF axis pathway by miR-155. Growth Factors (2017) 35:49–60. doi: 10.1080/08977194.2017.1345900 28683581

[28] Hikami K Kawasaki A Ito I Koga M Ito S Hayashi T. Association of a functional polymorphism in the 3’-untranslated region of SPI1 with systemic lupus erythematosus. Arthritis Rheum (2011) 63:755–63. doi: 10.1002/art.30188 21360505

[29] Patrick DM de la Visitación N Krishnan J Chen W Ormseth MJ Stein CM. Isolevuglandins disrupt PU.1 mediated C1q expression and promote autoimmunity and hypertension in systemic lupus erythematosus. JCI Insight (2022) 7:1–24. doi: 10.1172/jci.insight.136678 PMC9310530 35608913

[30] Ouyang H Shi Y Liu Z Feng S Li L Su N. Increased interleukin-9 and CD4+IL-9+ T cells in patients with systemic lupus erythematosus. Mol Med Rep (2013) 7:1031–7. doi: 10.3892/mmr.2013.1258 23291628

[31] Sheng Y Zhang J Li K Wang H Wang W Wen L. Bach2 overexpression represses Th9 cell differentiation by suppressing IRF4 expression in systemic lupus erythematosus. FEBS Open Bio (2021) 11:395–403. doi: 10.1002/2211-5463.13050 PMC7876501 33249782

[32] Umazume A Kezuka T Matsuda R Usui Y Takahashi H Yamakawa N. Role of PU.1 expression as an inflammatory marker in experimental autoimmune uveoretinitis. Ocul Immunol Inflammation (2018) 26:951–63. doi: 10.1080/09273948.2017.1299867 28448751

[33] Watt S Vasquez L Walter K Mann AL Kundu K Chen L. Genetic perturbation of PU.1 binding and chromatin looping at neutrophil enhancers associates with autoimmune disease. Nat Commun (2021) 12:1–12. doi: 10.1038/s41467-021-22548-8 33863903 PMC8052402

[34] Felton JM Bouffi C Schwartz JT Schollaert KL Malik A Vallabh S. Aiolos regulates eosinophil migration into tissues. Mucosal Immunol (2021) 14:1271–81. doi: 10.1038/s41385-021-00416-4 PMC8542574 34341502

[35] Gerlach K Hwang Y Nikolaev A Atreya R Dornhoff H Steiner S. TH9 cells that express the transcription factor PU.1 drive T cell-mediated colitis *via* IL-9 receptor signaling in intestinal epithelial cells. Nat Immunol (2014) 15:676–86. doi: 10.1038/ni.2920 24908389

[36] Shao Q Wang Y Liu Z Liu H Wang Y Zhao Y. Th9 cells in peripheral blood increased in patients with immune-related pancytopenia. J Immunol Res (2020) 2020. doi: 10.1155/2020/6503539 PMC7222599 32455141

[37] Alter-Koltunoff M Ehrlich S Dror N Azriel A Eilers M Hauser H. Nramp1-mediated innate resistance to intraphagosomal pathogens is regulated by IRF-8, PU.1, and miz-1. J Biol Chem (2003) 278:44025–32. doi: 10.1074/jbc.M307954200 12904288

[38] Chen Q Mang G Wu J Sun P Li T Zhang H. Circular RNA circSnx5 controls immunogenicity of dendritic cells through the miR-544/SOCS1 axis and PU.1 activity regulation. Mol Ther (2020) 28:2503–18. doi: 10.1016/j.ymthe.2020.07.001 PMC7646215 32681834

[39] Meng Y Li J Ye Z Yin Z Sun Q Liao Z. MicroRNA-148a facilitates inflammatory dendritic cell differentiation and autoimmunity by targeting MAFB. JCI Insight (2020) 5. doi: 10.1172/jci.insight.133721 PMC7205423 32213710

[40] Wang XQ Hu GH Kang HY Shen Y Ke X Hong SL. High frequency of t helper type 9 cells in chinese patients with allergic rhinitis. Asian Pacific J Allergy Immunol (2015) 33:301–7. doi: 10.12932/AP0609.33.4.2015 26708394

[41] Chowdhury K Kumar U Das S Chaudhuri J Kumar P Kanjilal M. Synovial IL-9 facilitates neutrophil survival, function and differentiation of Th17 cells in rheumatoid arthritis. Arthritis Res Ther (2018) 20:1–12. doi: 10.1186/s13075-017-1505-8 29382374 PMC5791733

[42] Chen Z Fan R Liang J Xiao Z Dang J Zhao J. NFIL3 deficiency alleviates EAE through regulating different immune cell subsets. J Adv Res (2021) 39:225–35. doi: 10.1016/j.jare.2021.10.011 PMC9263648 35777910

[43] Guggino G Pio La Manna M Di Liberto D Lo Pizzo M Grasso G Schinocca C. Interleukin 9 neutralisation reduces collagen-induced arthritis severity in mouse models. Clin Exp Rheumatol (2022) 1. doi: 10.55563/clinexprheumatol/chima7 35616583

